# Teaching case 2-2020: Lhermitte-Duclos disease in a female patient with Cowden syndrome 

**DOI:** 10.5414/NP301276

**Published:** 2020-02-12

**Authors:** Simon Hametner, Johannes A. Hainfellner, Martin Ortler, Christine Haberler

**Affiliations:** 1 Division of Neuropathology and Neurochemistry, Department of Neurology, Medical University of Vienna, and; 2 Department of Neurosurgery, Krankenanstalt Rudolfstiftung, Vienna, Austria, and Department of Neurosurgery, Medical University of Innsbruck, Innsbruck, Austria

**Keywords:** Cowden syndrome, Lhermitte-Duclos disease, PTEN

## Abstract

No abstract available.

We present a rare case of adult Lhermitte-Duclos disease (aLDD) in the setting of Cowden syndrome in a 37-year old female patient. Clinical history together with neuroimaging and neuropathological findings are presented. 

The patient was admitted to Neurosurgery because of blurred vision and severe headaches associated with nausea and vomiting, persisting for 2 weeks. She also reported tremor and paresthesias in the left upper extremity. A CT scan revealed a 6 × 5 × 3 cm space-occupying posterior fossalesion on the left side with distortion of the 4^th^ ventricle and signs of ipsilateral tonsillar herniation. Ophthalmologic examination revealed a marked bilateral papillary edema. A subsequently performed MRI scan confirmed a demarcated, striated lesion with alternating T1-isointense and hypointense bands (Figure 1A, B). Contrast enhancement was completely absent. The aqueduct was not occluded; however, the temporal horns of the lateral ventricles were slightly enlarged. The lesion was surgically resected. 

Neuropathological evaluation revealed small portions of adjacent normally configured cerebellar tissue and a marginally pleomorphic and slightly hypercellular tumor. The tumor replaced the cerebellar cortex in a band-like fashion and contained multiple gangliocytes with distinct nucleoli (Figure 1C, H & E staining). The underlying white matter was characterized by loosening of the tissue and very occasional scattered dysplastic gangliocytes. No mitotic figures were encountered in the tumor, and immunohistochemical staining for MIB1 marked only few and small nuclei (not shown). The band-like tumor was strongly positive for synaptophysin (Figure 1D) and contained multiple ectatic blood vessels, which could be clearly depicted by CD34 immunohistochemistry (Figure 1E). CD34 staining additionally revealed upregulated expression in glial cells within the tumour in a focal and bushy-like fashion, while the gangliocytes remained negative. Finally, immunohistochemistry for PTEN, which was absent in the enlarged neurons but preserved in the blood vessels (Figure 1F), corroborated the neuropathological diagnosis of a dysplastic cerebellar gangliocytoma and thus aLDD. 

aLDD is diagnostic for Cowden syndrome [1]. Reassessment of the patient’s clinical history revealed a follicular thyroid carcinoma and a ductal breast carcinoma in-situ. Moreover, review of the relevant literature confirmed the MRI study as highly typical for aLDD [2]. 

This case underscores the importance of clinical, imaging, and neuropathological synopsis in order to adequately diagnose and counsel the patient as well as monitor carcinoma recurrences or other manifestations in this patient, such as endometrial carcinoma. 

## Funding 

No funding received for this study. 

## Conflict of interest 

The authors declare no conflicts of interest. 

**Figure 1:**
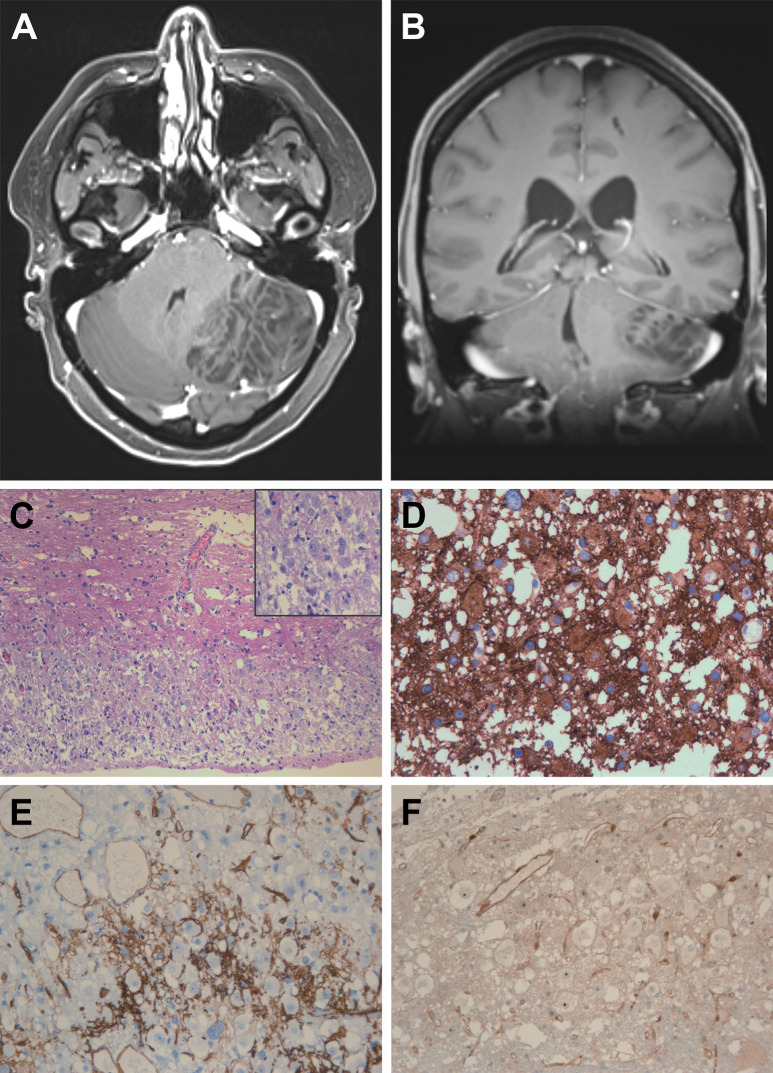
Post-contrast axial (A) and coronal (B) T1-weighted image of the space-occupying lesion. C: H & E stained section of the band-like superficial hamartomatous lesion with multiple gangliocytes showing distinct nucleoli (inset); D: Synaptophysin IHC shows strong reactivity of the tumorous neuropil matrix; E: CD34 IHC reveals reactive glial upregulation in a bushy-like fashion. In addition, multiple ectatic blood vessels are highlighted; F: PTEN IHC with preserved endothelial staining and negativity of gangliocytes.

## References

[1] Zhou XP Marsh DJ Morrison CD Chaudhury AR Maxwell M Reifenberger G Eng C Germline inactivation of PTEN and dysregulation of the phosphoinositol-3-kinase/Akt pathway cause human Lhermitte-Duclos disease in adults. Am J Hum Genet. 2003; 73: 1191–1198. 14566704 10.1086/379382 PMC1180498

[2] Wei G Zhang W Li Q Kang X Zhao H Liu X Tang X Wu Y Han J Yin H Magnetic resonance characteristics of adult-onset Lhermitte-Duclos disease: An indicator for active cancer surveillance? Mol Clin Oncol. 2014; 2: 415–420. 24772310 10.3892/mco.2014.258 PMC3999135

